# The impact of focused federal initiatives on pediatric cancer research

**DOI:** 10.1002/cncr.70172

**Published:** 2025-11-14

**Authors:** Subhashini Jagu, Malcolm A. Smith, Nita L. Seibel, Emily S. Tonorezos, Brigitte C. Widemann, Holly A. Gibbons, Mary K. Holohan, Ziky A. Ababiya, Jaime M. Guidry Auvil, Douglas R. Lowy, James H. Doroshow, Anne E. Lubenow, Gregory H. Reaman

**Affiliations:** ^1^ National Cancer Institute Bethesda Maryland USA; ^2^ Weill Cornell Medicine New York New York USA

**Keywords:** biospecimens, CCDI, clinical sequencing, data science, data science: database management systems, data sharing, funding, pediatric cancers, policy, repositories, STAR Act

## Abstract

Federal initiatives, including the Childhood Cancer Data Initiative and the Childhood Cancer Survivorship, Treatment, Access, and Research Act, are advancing pediatric cancer data sharing and strengthening infrastructure required for clinical trials, biobanking, and biospecimen collection. These efforts aim to transform pediatric cancer research to ultimately improve treatment outcomes, quality of life, and survivorship.

## INTRODUCTION

Research progress in recent decades has led to improved outcomes for children, adolescents, and young adults (AYAs) with cancer, but cancer remains the leading cause of disease‐related death in children in the United States.^1^, ^2^, ^3^, ^4^ Moreover, more than 30% of successfully treated children experience potentially debilitating and life‐threatening late effects of treatment,^5^, ^6^, ^7^ affecting quality of life. The lower incidence of cancers in children when compared with cancers in adults poses significant challenges to the design and conduct of clinical trials. Historically, pediatric cancer research has faced challenges such as limited market incentives for pharmaceutical development, low industry engagement, and insufficient data‐sharing infrastructure, hindering the development of new therapies.

To address longstanding challenges in investment, survivorship, cancer registry infrastructure, access to care, data collection, and research, and in response to vigorous pediatric cancer advocacy efforts, Congress passed the Childhood Cancer Survivorship, Treatment, Access, and Research (STAR) Act in 2018. The legislation was reauthorized in 2023 with overwhelming bipartisan support. The STAR Act and its reauthorization authorize $30 million annually to enhance research in three areas of focus: pediatric cancer biospecimen collection and biobanking, childhood and AYA survivorship research, and pediatric cancer registry efforts. The National Cancer Institute (NCI) leads implementation of provisions focused on biobanking and survivorship research, and the Centers for Disease Control and Prevention leads implementation of the registry provisions, as authorized in the law. The STAR Act directs these Department of Health and Human Services agencies to support expanding biobanking and biospecimen collection, improving the quality of life for childhood cancer survivors, and expanding registry programs to improve early case capture and tracking of childhood and AYA cancer survivors.

In parallel, NCI launched the Childhood Cancer Data Initiative (CCDI), a 10‐year, $50‐million‐per‐year federal investment announced in 2019 as a special initiative proposed in the President’s fiscal year 2020 Budget Request.^8^ The goals of CCDI are to collect data from every child and AYA diagnosed with cancer; establish a national strategy for clinical and molecular characterization of pediatric cancers to improve accuracy of diagnosis and inform treatment; and develop platforms and tools that integrate clinical, registry, and research data to enhance etiology and prevention efforts and support the development of new treatment options.

The U.S. Congress, in a bipartisan manner, has supported the goals of the STAR Act and CCDI, maintaining appropriations at the authorized level of $30 million per year for the STAR Act and the requested $50 million per year for CCDI. Together, biospecimen acquisition, processing, and storage supported through the STAR Act and data generation, harmonization, improved interoperability, analysis, and sharing enabled by CCDI, work synergistically to advance progress across both initiatives.

These efforts are helping ensure that the benefits of precision medicine reach all children and families affected by cancer, while advancing our understanding of the biological and clinical complexity of childhood cancer.

## THE STAR ACT: BIOSPECIMEN COLLECTION AND IMPROVED QUALITY OF LIFE FOR SURVIVORS

The Children’s Oncology Group (COG), the pediatric component of NCI's National Clinical Trials Network, operates across more than 200 sites.^9^ Eighty percent of children diagnosed with cancer in the United States are treated at a COG member institution.^10^ Each year, COG enrolls thousands of patients in a wide range of studies, including clinical trials for newly diagnosed and relapsed patients, early‐phase trials of novel and supportive care agents, and numerous nontherapeutic studies, such as epidemiology and registry efforts. Through funding from NCI to implement the STAR Act, the COG has expanded its collection of tissue and blood samples. These efforts allow focused investigation on tumor types for which treatments often fail. It enables COG studies to collect samples at diagnosis (to determine clinical trial eligibility) and at relapse, thereby enabling investigations to understand treatment failure and evaluate novel therapies for relapsed disease with implications on the future design of more effective clinical trials of innovative therapies targeting hard‐to‐treat diseases. Since 2020, more than 5000 biospecimens (including extracted nucleic acids) and more than 10,000 digitized pathology images have been shared with 22 investigators across 31 distinct projects.

STAR Act funding has played a critical role in expanding the Childhood Cancer Survivorship Study (CCSS),^11^ the world’s largest open survivorship research resource. With >38,000 eligible survivors diagnosed between 1970 and 1999 and >25,000 contributing health and quality‐of‐life data, CCSS provides detailed treatment annotations, longitudinal follow‐up, and a biorepository that includes tissue from >11,000 subsequent malignancies and sequencing data for nearly 10,000 survivors. STAR Act support has enabled the addition of methylation data, clonal hematopoiesis assessments, and expanded sequencing among survivors with chronic health conditions. Data will be released through the CCDI Data Ecosystem, providing investigators with access to comprehensive longitudinal data from completion of therapy through survivorship. The Act also provides a mechanism to extend the cohort to include survivors diagnosed between 2000 and 2025, allowing the study of long‐term effects of more contemporary therapies and novel agents.

In addition to supporting biospecimen collection and storage for future research use, the STAR Act stipulated expanded efforts to improve long‐term outcomes for survivors of childhood cancers through its support of more than 30 new survivorship research grants in response to Requests for Applications aligned with STAR Act research areas of emphasis, including: improving survivorship care,^12^ improving outcomes and long‐term quality of life,^13^ and addressing barriers to health care transitions for childhood and AYA survivors of cancers. NCI also commissioned three Agency for Healthcare Research and Quality evidence reviews on childhood cancer survivorship—addressing transitions in care,^14^ models of care,^15^ and barriers to care.^14^

A key challenge addressed by provisions in the STAR Act is the delay in cancer case reporting, which can take 2 or more years. To help overcome this, the Centers for Disease Control and Prevention was authorized to provide grants for state cancer registries to improve the reporting and tracking of childhood and AYA cancers, facilitating data integration and improved research efficiency. These grants help collect information on cancer cases faster through improved electronic reporting and other infrastructure developments.

## CCDI: DEVELOPING INFRASTRUCTURE TO ACCELERATE DATA SHARING AND USE

Pediatric cancers are relatively rare, and facilitating access to large, harmonized data sets is crucial for advancing research. Integrating diverse types of data across multiple sources enables researchers to uncover new disease mechanisms, discover potentially actionable targets, develop targeted therapies, and identify genetic markers of cancer predisposition and molecular vulnerabilities to better predict patient outcomes.

However, comprehensive analyses are often limited by data fragmentation^16^ across clinical trial data management systems and data sets, electronic health records, state and regional cancer and disease‐specific registries, laboratory reports, imaging archives, and genomic data sets. This siloing presents significant challenges for clinicians and researchers who rely heavily on integrated data to inform decision‐making.

To meet the need for unified data access and sharing, CCDI established a robust data infrastructure: the CCDI Data Ecosystem.^17^, ^18^ The CCDI Data Ecosystem integrates multimodal preclinical and participant‐level clinical, registry, real world–sourced, genomic, transcriptomic, and epigenomic data from multiple pediatric cancer projects to improve statistical power, expand investigator access, and foster multi‐institutional collaboration to further enhance data collection and sharing and ultimately decrease the global burden of cancer. The ecosystem includes interoperable, highly accessible platforms and tools developed by CCDI that researchers can use to find, access, and analyze data.^1^, ^17^ These resources are accessible through the CCDI Hub, an entry point for researchers, data scientists, and community scientists looking to use and connect with CCDI‐supported data, tools, and applications. Key components of the CCDI Data Ecosystem include the:

- cBioPortal Cancer Data Explorer, a platform that allows users to search by gene, mutation, cancer type, and more, and visualize complex genomic data without downloading large data sets;
- CCDI Data Federation Resource, an application programming interface that allows researchers to query data across multiple sources as if they were in a unified database;
- CCDI Participant Index, an application programming interface that enables mapping and management of de‐identified participant IDs across studies to support longitudinal and cross‐study analyses;
- Childhood Cancer Clinical Data Commons, a platform that offers harmonized demographic and phenotypic clinical data through an open‐access interface;
- Childhood Cancer Data Catalog, a curated inventory of pediatric cancer data resources;
- Explore Dashboard, an integrated CCDI Hub tool that provides participant‐centric search functionality by connecting participants with files and samples. The Explore Dashboard enables researchers to find data within a single study or across multiple studies and create cohorts based on filtered metrics of interest (i.e., demographics, diagnosis, samples); and the
- National Childhood Cancer Registry, a rapidly expanding public health resource providing population‐level data on childhood and AYA cancer incidence, treatment, and outcomes.

The CCDI Data Ecosystem has the potential to enable large‐scale, data‐driven discoveries in pediatric oncology, helping to define molecular subtypes, identify actionable biomarkers and therapeutic targets, and develop validated predictive models that inform clinical trial design and improve survivorship outcomes. In the past 12 months, CCDI resources and educational web pages have attracted nearly 44,500 visitors, and the CCDI Hub received more than 5000 visitors, demonstrating growing engagement from the pediatric cancer research community. CCDI continues to strengthen its data infrastructure by integrating multimodal data to create artificial intelligence–ready resources.

## BUILDING THE FOUNDATION FOR PRECISION PEDIATRIC ONCOLOGY: CCDI AND STAR ACT SUPPORT FOR THE MOLECULAR CHARACTERIZATION INITIATIVE

Launched by NCI in 2022, the Molecular Characterization Initiative (MCI)^19^ provides comprehensive molecular profiling for newly diagnosed children and AYAs with central nervous system tumors, soft tissue sarcomas, rare pediatric cancers, high‐risk neuroblastomas, and metastatic Ewing sarcoma treated at COG–affiliated institutions. MCI uses COG’s Project:EveryChild (APEC14B1) protocol to enroll participants, collect specimens, and annotate clinical data. This foundational registry and screening platform integrates eligibility assessment, molecular characterization, biology studies, and outcome tracking to inform clinical trial enrollment and support national research efforts.

Many participating sites previously lacked access to this level of testing. MCI enables rapid return (14 days from time of specimen receipt) of clinical molecular profiling to treating clinicians—at no cost to patients or families—and supports timely and informed treatment decisions and clinical trial matching. To date, MCI has generated more than 15,000 sequencing assays from more than 7000 participants, revealing somatic mutations, gene fusions, actionable targets, and germline variants. Importantly, these results have been made available in near real time to treating physicians and have refined diagnosis and informed treatment strategies using molecularly targeted agents in 30% and 12% of patients, respectively. In addition, the MCI is now pivotal in eligibility determination for many COG clinical trials. Approximately 13% of participants were found to have a germline cancer predisposition, emphasizing the importance of tumor–normal profiling for genetic counseling and long‐term surveillance.

CCDI and the STAR Act collaboratively enable the operational capabilities of MCI. The STAR Act provides infrastructure support for biospecimen acquisition, processing, and banking, including nucleic acid extraction, sample distribution, and quality control. CCDI supports specimen characterization, data integration, and public sharing through the CCDI Data Ecosystem,^1^ which houses de‐identified sequencing results and clinical reports, along with the linked demographic, diagnosis, and treatment data to accelerate discovery and improve outcomes for children and AYAs with cancer.

## FUTURE PROSPECTS

To maintain momentum, dedicated initiatives to accelerate pediatric cancer research progress require sustained investment and community engagement to support infrastructure and resource development, long‐term data collection and harmonization, and biospecimen collection. CCDI heavily relies on the community and coordinates efforts across multiple independent administrative and research systems. However, this approach also demands thoughtful communication and coordination strategies to ensure efficiency, identification, and resolution of potential or perceived conflicts of interest.

Sustained investment in CCDI and the STAR Act will enable expanded molecular characterization efforts and lay the groundwork for a broader national strategy that improves diagnosis, treatment, and ultimately survivorship across all pediatric cancer populations. These efforts also help define parameters for third‐party payer coverage of molecular testing, ensuring continued access regardless of where patients receive care. Continued support will allow for the inclusion of individuals treated outside of COG institutions, and those with relapsed or refractory disease. It will also support the development of disease‐specific molecular assays and is already enabling the use of banked biospecimens to fuel discovery science.

Additionally, continued support through CCDI and the STAR Act will enable the expansion of molecular characterization efforts for at‐risk family members to further investigations of genetic epidemiology and cancer etiology, laying the foundation for future prevention studies in high‐risk children and presumably unaffected siblings. It will expand access to informed genetic counseling and educational services, enhance data integration, and advance artificial intelligence–driven analyses to accelerate discoveries leading to new therapeutic agent development. Sustained investment will also enable the recruitment of more recent survivor cohorts (diagnosed from 2000 onward), which will allow researchers to study the long‐term effects of newer therapies (e.g., immunotherapies, molecularly targeted treatments, radiation approaches) and design future clinical trials.

## CONCLUSION

The CCDI Data Ecosystem, MCI, the expansion of the CCSS resource, and other NCI programs funded through CCDI and the STAR Act are unique but complementary initiatives. Together, they aim to advance data sharing, transform the genetic epidemiology of childhood cancer, and identify new genomic vulnerabilities that can lead to more effective treatments. These efforts also support state‐of‐the‐art clinical trials through the application of molecular diagnostics and ultimately improve treatment outcomes, quality of life, and survivorship. They also strengthen infrastructure for biobanking and biospecimen collection, especially for rare tumor types, increasing sample availability and facilitating research on these cancers with generally poor outcomes. Therefore, continuous support for both CCDI and the STAR Act has been critical for accelerating progress in childhood cancer.

## AUTHOR CONTRIBUTIONS

The contributions of the National Institutes of Health (NIH) author(s) are considered Works of the United States Government. The findings and conclusions presented in this paper are those of the author(s) and do not necessarily reflect the views of the NIH or the U.S. Department of Health and Human Services.

## CONFLICT OF INTEREST STATEMENT

The authors declare no conflicts of interest.

## Data Availability

Data sharing not applicable to this article as no datasets were generated or analysed during the current study.
